# 

**Published:** 1828-09

**Authors:** 


### TO SUBSCRIBERS.

---

The great delay in the publication of this number requires an explanation. On the 18th of September, the day on which the printing of the number was commenced, the Editor, in rescuing one of his friends from the flames, suffered extensive burns on both his hands. The accident was followed by various afflicting constitutional disorders, from which at this time, October 24, he is but beginning to recover. He was thus prevented from finishing several original articles for the number, and has been compelled to substitute selected matter; which he hopes, however, from its intrinsic excellence, will not be unacceptable to his readers.

The October number will positively be committed to the press in a few days, and issued with all possible expedition.

---

### MEDICAL SOCIETY.

The semi-annual meeting of the *First District Medical Society of Ohio*, will be held in Cincinnati at the Medical College Edifice, *on the last Tuesday of November*, 1828, commencing at 10 o'clock in the morning.

JAMES M. MASON, M. D.
*Recording Secretary.*

